# Does curcumin supplementation affect inflammation, blood count and serum brain-derived neurotropic factor concentration in amateur long-distance runners?

**DOI:** 10.1371/journal.pone.0317446

**Published:** 2025-01-14

**Authors:** Sebastian Bańkowski, Ziemowit Bronisław Wójcik, Małgorzata Grabara, Dariusz Ozner, Tomasz Pałka, Agata Stanek, Ewa Sadowska-Krępa

**Affiliations:** 1 Institute of Sport Sciences, Jerzy Kukuczka Academy of Physical Education in Katowice, Katowice, Poland; 2 Faculty of Health Sciences in Katowice, Medical University of Silesia, Katowice, Poland; 3 Department of Physiology and Biochemistry, Faculty of Physical Education and Sport, University of Physical Education in Krakow, Krakow, Poland; 4 Department of Internal Medicine and Metabolic Diseases, Faculty of Health Sciences in Katowice, Medical University of Silesia in Katowice, Upper-Silesian Medical Centre of the Medical University of Silesia in Katowice, Katowice, Poland; Alexandria University, EGYPT

## Abstract

Curcumin is known for its potential health benefits; however, the evidence remains inconclusive regarding its necessity as a supplement for athletes during the preparatory phase of training. This study aimed to assess the effect of 6-week curcumin supplementation at a dose of 2g/day on selected inflammatory markers, blood count, and brain-derived neurotropic factor (BDNF) levels in middle-aged amateur long-distance runners during the preparatory period of a macrocycle. Thirty runners were randomly assigned to either a curcumin-supplemented group (CUR, n = 15) or a placebo group (PLA, n = 15). Venous blood samples were collected at rest, immediately post-exercise, and 1h post-exercise. The participants underwent a graded exercise stress test, with an increasing inclination angle after reaching a speed of 14 km/h, both before and after the 6-week supplementation period. Blood samples were collected at rest, 3 minutes post-stress test, and after 1 hour of recovery. The results showed no significant changes in C-reactive protein (CRP), interleukin-6 (IL-6), tumor necrosis factor α (TNF-α), interleukin-1 β (IL-1β), or blood morphology due to curcumin supplementation. However, BDNF levels increased by 21% in the CUR group post-supplementation, while a 5% decrease was observed in the PLA group. These findings do not support a significant effect of curcumin supplementation on inflammatory markers, blood count, or BDNF concentration. Further research is warranted to determine the potential benefits of curcumin supplementation for endurance athletes during the preparatory period for a training cycle.

## Introduction

Natural plant supplements are widely used by endurance athletes to alleviate exercise-induced oxidative stress, accelerate recovery, and enhance performance [1]. Curcumin, a natural polyphenol, is one such supplement, that has gained popularity in the athletic community.

Curcumin is derived from the root of *Curcuma longa*, a member of the ginger family commonly used in traditional medicine and oriental cuisine as a spice (e.g., curry) [2, 3]. Structurally, curcumin is an amphiphilic compound with predominantly hydrophobic domains [2, 3]. Research suggests that curcumin supplementation may help reduce inflammation induced by physical exercise [2–4]. Endurance exercise has been shown to elevate the levels of pro-inflammatory cytokines in the bloodstream, such as interleukin-1β (IL-1β) and interleukin-6 (IL-6), in the post-exercise period [5]. IL-1β plays a crucial role in immune response activation, promoting lymphocyte activity, stimulating macrophages, enhancing leukocyte/endothelial adhesion, and driving the liver to release acute-phase proteins like C-reactive protein (CRP) [6, 7].

Curcumin’s anti-inflammatory effects are attributed to its ability to modulate key inflammatory signaling pathways, including the nuclear factor-κB (NF-κB), Janus kinase/signal transducer and activator of transcription (JAK/STAT), and mitogen-activated protein kinase (MAPK) pathways [8]. Curcumin exerts its anti-inflammatory properties by inhibiting NF-κB activation, JAK/STAT phosphorylation, and MAPK signaling, thereby reducing the release of inflammatory mediators such as tumor necrosis factor α (TNFα) and IL-6 at the injury [9].

Additionally, curcumin exhibits other health-promoting properties, such as supporting immune function and nervous system health. Its neuroprotective effects are of particular interest, as they help prevent the formation of pathological proteins associated with neurodegenerative diseases [10–15]. It is well known that regular, long-term endurance exercise influences blood morphology [16–18], including increasing levels of brain-derived neurotropic factor (BDNF) [19–21]. Aerobic exercise enhances oxygen transport capacity by increasing the total hemoglobin content and the number of red blood cells (RBC) [22], and may also modulate immune functions by altering the levels of circulating leukocytes, particularly lymphocytes (LYM) and neutrophils (NEUT) [18].

As a potent phytochemical, curcumin may influence hematological parameters by stimulating erythropoiesis and leukocytosis, while regulating immune system activity [23]. Inflammation is often assessed using ratios such as the neutrophil-to-lymphocyte ratio (NLR), platelet-to-lymphocyte ratio (PLR), and monocyte-to-lymphocyte ratio (MLR), which serve as sensitive biomarkers for inflammatory status [24].

BDNF is involved in various physiological processes, including neuronal development, learning and memory [19–21]. Given curcumin’s neuroprotective properties, it is plausible that supplementation could influence the expression of this neurotrophin [25].

The limited number of studies [26–28] investigating the effects of curcumin supplementation in long-distance runners over extended periods (longer than a few days) at a daily dose of 2 g, particularly concerning its impact on biochemical markers of immune response and neuroprotection, prompted this study. It should be emphasized that most previous studies have focused on the effect of curcumin supplementation on inflammation markers (CRP, IL-1β, IL-6, TNFα) and BDNF at doses ranging from 150 to 1500 mg daily over 4 to 6 weeks [8, 15, 29–32]. While higher doses of curcumin (5–6 g/day) have been suggested to enhance bioavailability, a dose of 2 g/day is considered safe, with minimal risk of side effects such as diarrhea, rashes, and headache [33–35].

Therefore, the aim of this study was to assess whether 6-week curcumin supplementation at a dose of 2 g per day would influence inflammation, BDNF levels, and blood morphology in middle-aged amateur long-distance runners following an incremental treadmill running test during the preparatory period of the training cycle. It was hypothesized that curcumin supplementation would significantly reduce inflammation and alter BDNF concentrations and peripheral blood morphology in response to exercise stress test.

## Materials and methods

### Participants

The sample size was calculated using G*Power version 3.1.9.7, developed by Heinrich Heine University in Düsseldorf, Germany. An effect size of 0.5 was assumed, with an alpha error probability of 0.05 and a test power of 0.84. Based on these indices, the required sample size was determined to be thirty.

**Fig 1:**
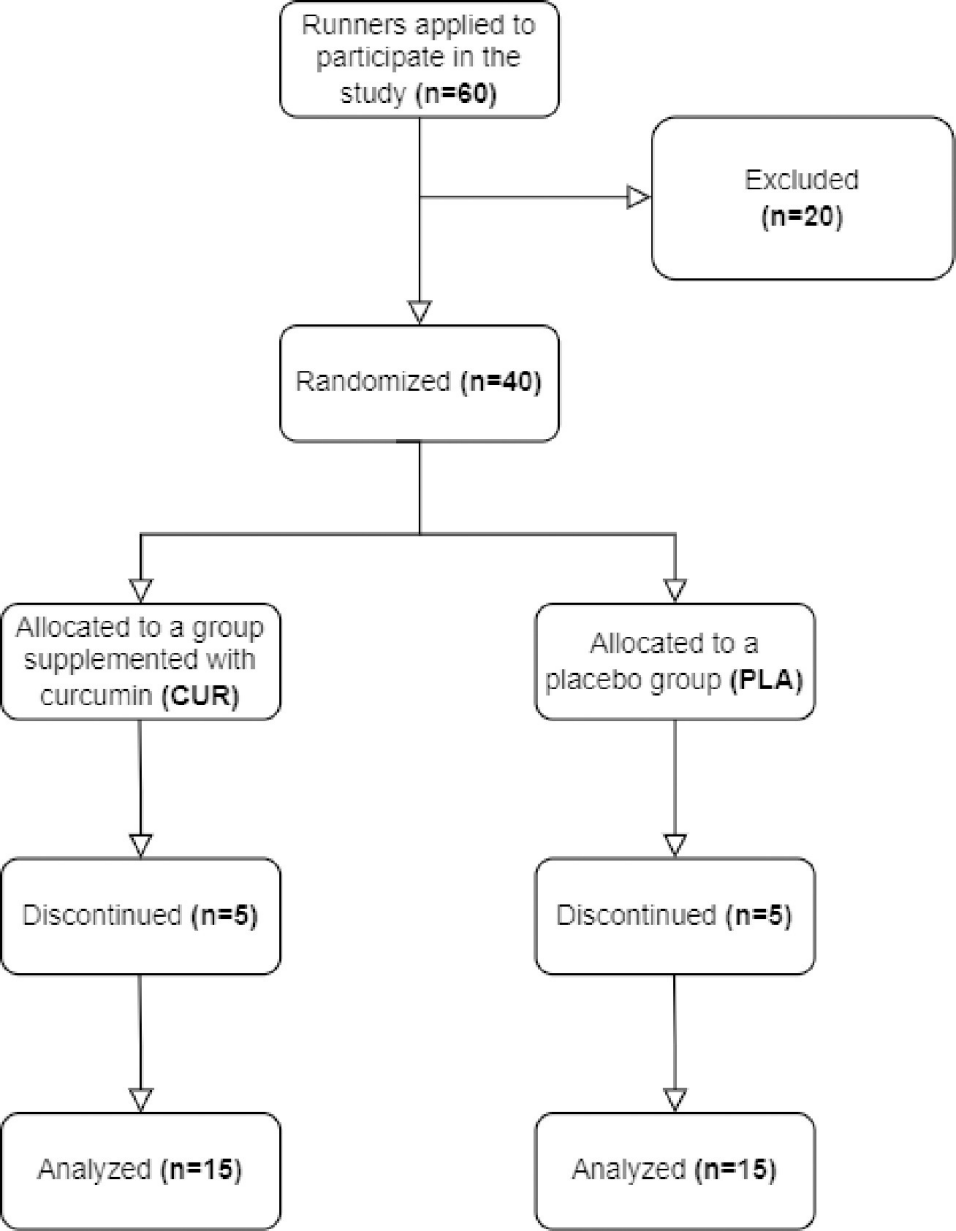
Participants flow chart.

Sixty middle-aged amateur long-distance runners volunteered for this study. Twenty participants were excluded due to insufficient training running experience, and an additional ten did not complete the study. Consequently, the final study group consisted of thirty amateur long-distance runners, aged 38.33 ± 5.28 years (Fig 1).

Exclusion criteria included tobacco use, alcohol consumption, intake of any medications, non-steroidal anti-inflammatory drugs (NSAIDs), or dietary supplements within the four weeks prior to the study. Inclusion criteria required participants to be an adult males with a minimum of three years of running training experience.

This study was conducted as a double-blind, placebo-controlled, randomized trial. All participants were informed of the study aim and nature and provided their written informed consent. The study protocol conformed to the ethical guidelines of the World Medical Association Declaration of Helsinki and was approved by the Institutional Ethics Committee (certificate no. 11/2019). Additionally, the study was retrospectively registered in the Australian New Zealand Clinical Trials Registry (ANZCTR) under the number ACTRN12622000456752. The delay in study registration, which occurred after the commencement of participant enrollment, was primarily due to the need to access this unique study group. All participants were only available for inclusion during the preparatory period of their training cycle, which took place in November. As a result, recruitment and follow-up occurred between 18 November 2019 and 12 February 2020. Data were collected at Academy of Physical Education in Katowice (Poland). The authors confirm that all ongoing and related trials for this intervention are registered.

Participants were randomized into two groups for this study protocol: 1) a control placebo group (PLA, *n*  =  15) and 2) a group supplemented with curcumin extract (CUR, n  =  15, 2 g/day for six weeks). The intake of supplements by both the PLA and CUR groups was monitored. The basic physical characteristics of participants are presented in Table 1.

**Table 1:** Basic characteristics of participants.

Variable	Trial	PLA (n = 15)	CUR (n = 15)
		M±SD	M±SD
Age (years)		39.6±5.8	37.1±4.5
Height (cm)		177.4±6.3	178.3±7.9
Weight (kg)		76.7±5.5	74.4±11.1
BMI (kg/m^2^)		24.4±1.9	23.3±2.1
VO_2max_ (ml/min/kg)	1^st^	50.8±4.4	49.9±5.3
VO_2max_ (ml/min/kg)	2^nd^	52.4±5.4	52.0±0.7
Experience (years)		4.0±0.9	4.3±1.0

Notes: M-mean; SD–standard deviation; PLA—placebo group; CUR–curcumin supplemented group

### Exercise test and blood collection

All participants completed an incremental treadmill running test (a Cosmed treadmill, Germany) while connected to a breath-by-breath gas analyzer (MetaLyzer 3B-R2, Leipzig, Germany) to determine maximal oxygen uptake (VO_2_max). This test was conducted twice: before the start of the supplementation period (1st trial) and after 6 weeks of supplementation with either placebo or curcumin (2nd trial). The treadmill speed was increased by 2 km/h every 3 minutes until a running speed of 14 km/h was reached. Afterward, the incline was increased by 2.5° every 3 minutes until the participants reached exhaustion. Training plans were based on Daniels’ running formula [36].

During the preparatory phase of the training cycle, participants ran an average distance of 104.37 ± 13.30 km per week (range: 85km/week-130km/week) at a pace of 4.83 ± 0.32 min/km (min-max: 4.22 min/km—5.02 min/km). In each trial, venous blood samples were collected from the cubital vein at three time points: at rest, then at 3 min post-test, and after 1 hour of recovery. Blood was drawn into vacuum tubes (*BD Vacutainer™ Serum Tube*, *UK)* containing a serum clot activator (CAT). The tubes were left to stand for 30 min to allow clotting, and serum was then separated by centrifugation for 10 min at 1000 × g at 4˚C (SIGMA 2-16KL, Sigma Laborzentrifugen GmbH, Germany). The samples were subsequently frozen and stored at −80°C for no longer than one month, without any freeze-thaw cycles.

### Supplement

Supplements were administered in the form of soft gelatin capsules (Nanga, Złotów, Poland) at a daily dose of 2 g curcumin extract for six weeks. Participants took two capsules after breakfast and two after dinner, accompanied by a glass of water. Each supplement capsule contained 500 mg turmeric extract standardized to contain 95% of curcumin, 10 mg of black pepper extract standardized to 95% piperine, and a shell composed of gelatine and purified water (13–17%). The placebo capsules contained 400 mg of corn-starch, 10 mg of riboflavin pigment, and a shell composed of gelatine and purified water (13–17%).

### Biochemical analyses

Venous blood samples were collected before the start and on the day following the completion of the supplementation period with either curcumin or placebo. The peripheral blood morphology was measured at the certified external laboratory. The Sysmex analyzer was used to assess blood morphology parameters, including red blood cells (RBC) count, hemoglobin (HGB), white blood cells (WBC) count, platelets (PLT) count, eosinophils (EOS) count, basophils (BASO) count, and percentages of neutrophils (NEUT%), lymphocytes (LYM%), immature granulocytes (IG%).

The serum concentration of C-reactive protein (CRP) was determined using a spectrophotometric method at the external laboratory, employing CRP-Turbilatex from Spinreact (MD1107101, Girona, Spain). The intra- and inter-assay coefficients of variation (CV) were not specified by the manufacturer. Serum concentrations of IL-1β, IL-6 and TNFα were measured by the immunoenzymatic assay using commercially available BioVendor kits (Czech Republic; RD194559200R, RD194015200R and RAF128R respectively). The inter-assay CV for IL-1β was 5.2%, with no information on the intra-assay CV. The intra- and inter-assay CVs for IL-6 were 5.1% and 4.7%, respectively, and for TNFα, they were 6,0% and 7,4%. The serum concentration of BDNF was measured using a BDNF kit (ELH-BDNF-1, RayBiotech, USA), with intra- and inter-assay CVs of < 10% and < 12%, respectively.

Based on the blood count results (PLT LYM, MON, and NEUT counts),), the following ratios were calculated: neutrophil-to-lymphocyte ratio (NLR), platelet-to-lymphocyte ratio (PLR), and monocyte-to-lymphocyte ratio (MLR).

All biochemical tests were conducted in accordance with PN-ENISO 9001:2015 (certificate no. PW19912-18B) and the manufacturers’ instructions by a certified biochemistry laboratory.

### Statistical analysis

The data are presented as means values and standard deviations (M ±SD). The normality of distributions and homogeneity of variance were tested using the Shapiro-Wilk test and Levene’s test, respectively.

The significance of differences between the curcumin-supplemented group and the placebo group was analyzed using the independent samples t-test or the Mann–Whitney U-test. The significance of differences between variables measured during the first and second trials was assessed using the paired-samples t-test or the Wilcoxon signed-rank test. Differences among the three time points (at the rest, 3 min post-test and 1 h post-test) were evaluated using the Friedman rang test with Kendall’s coefficient of concordance.

Effect sizes were calculated using Cohen’s d (for the t test), the r index (for the Wilcoxon signed-rank test), and Kendall’s W (for the Friedman rank test). The interpretation of effect sizes for Cohen’s d and Kendall’s W values was as follows: small effect 0.2- <0.5, medium effect 0.5- <0.8, and large effect ≥0.8. For the r index, the effect size was interpreted as follows: small effect 0.1- <0.3, medium effect 0.3- <0.5, and large effect ≥0.5.

The significance level for all tests was set at α = 0.05. Statistical analysis was performed using IBM SPSS Statistics 27.0 (PS Imago Pro 7.0)."

## Results

### Inflammation and BDNF concentration

The results of the biochemical analysis of inflammatory markers (CRP, IL-6, IL-1β, TNFα) and BDNF are detailed in Table 2. The inflammatory markers were the primary outcomes.

**Table 2:** Changes in selected inflammatory markers and BDNF concentration after curcumin supplementation.

Variable	Group	Trial	Rest	3 min post test	1 h post test
CRP (mg/l)	CUR	1^st^	0.59±0.47	0.53±0.52	0.56±0.39
		2^nd^ Δ	0.86±1.20 0.27±1.29 (87%)	0.89±1.17 0.35±1.67 (342%)	0.78±1.07 0.23±1.2 (260%)^c^
	PLA	1^st^	0.42±0.43	0.37±0.38	0.36±0.48
		2^nd^ Δ	1.16±1.01^aa^ 0.73±0.92 (381%)	1.25±0.97^a^ 0.87±1.03 (604%)	1.12±0.85^a^ 0.76±0.98 (495%)^c^
IL-6 (pg/ml)	CUR	1^st^	1.02±0.10	1.47±1.33	1.17±0.57
		2^nd^ Δ	1.06±0.06 0.04±0.12 (4%)	1.23±0.49 -0.24±1.37 (5%)	1.19±0.43 0.02±0.60 (10%)
	PLA	1^st^	1.00±0.04	1.33±1.09	1.31±0.53
		2^nd^ Δ	1.18±0.34^aa^ 0.18±0.35 (18%)	1.31±0.55 -0.02±1.12 (17%)	1.08±0.12 -0.24±0.56 (-7.9%)
IL-1β (pg/ml)	CUR	1^st^	0.90±0.03	0.89±0.04	0.90±0.04
		2^nd^ Δ	0.97±0.07^aa^ 0.07±0.08 (8%)	0.97±0.05^aaa^ 0.08±0.06 (9%)	0.98±0.05^aaa^ 0.08±0.06 (9%)^c^
	PLA	1^st^	0.97±0.16	1.26±1.30	0.91±0.03
		2^nd^ Δ	1.12±0.38^abb^ 0.15±0.31 (15%)	1.02±0.27^bb^ -0.24±1.36 (6%)	0.95±0.03^aaabb^ 0.04±0.03 (5%)^c^
TNFα (pg/ml)	CUR	1^st^	0.99±0.25	0.97±0.24	1.00±0.28
		2^nd^ Δ	1.00±0.41 0.01±0.44 (4%)	1.02±0.36^cc^ 0.05±0.37 (8%)^cc^	0.99±0.45 -0.01±0.56 (7%)^c^
	PLA	1^st^	1.12±0.49	1.05±0.37	1.05±0.40
		2^nd^ Δ	0.76±0.41^ab^ -0.35±0.50 (-22%)	0.66±0.27^aabcc^ -0.39±0.49 (25%)^cc^	0.70±0.43^b^ -0.35±0.58 (7%)^c^
BDNF (ng/ml)	CUR	1^st^	14.43±4.43^bbbc^	18.87±3.16^bbb^	14.56±3.83^bbb^
		2^nd^ Δ	15.68±3.46^bbb^ 1.24±4.20 (21%)	17.79±2.57^bbb^ -1.08±2.97 (-4%)	14.57±2.54^bbb^ 0.00±4.56 (14%)
	PLA	1^st^	17.80±3.75^bbbc^	20.09±3.41^bbb^	16.12±1.87^bbb^
		2^nd^ Δ	16.73±4.20^b^ -1.07±3.93 (-5%)	19.34±4.18^b^ -0.75±4.33 (-2%)	15.83±4.01^b^ -0.29±2.71 (-3%)

Note: CUR–group supplemented with curcumin; PLA–placebo; CRP–c-reactive protein; IL-6 –interleukin 6; IL-1β–interleukin 1β; TNFα–tumor necrosis factor alpha; BDNF—brain-derived neurotrophic factor; Δ–difference between 2^nd^ and 1^st^ measurements; significant differences: ^a^p<0.05, ^aa^ p<0.01, ^aaa^p<0.001 vs. respective basal values before treatment; ^b^p<0.05, ^bb^p<0.01, ^bbb^p<0.001 between respective values at rest, 3 min post-test and 1 h post-test; ^c^p<0.05, ^cc^p<0.01 between groups (CUR vs. PLA).

After the 6-week curcumin supplementation period, no significant changes in CRP concentrations were detected compared to baseline levels. In contrast, the PLA group exhibited a significant increase in CRP concentration at rest (Z = -2.62, p = 0.009, r = 0.68), at 3 minutes post-test (Z = -2.45, p = 0.014, r = 0.63), and at 1 hour post-test (Z = -2.41, p = 0.016, r = 0.62) during the second trial, indicating a large effect size when compared to the first trial values.

In the CUR group, IL-1β levels significantly increased during the second trial at rest (Z = -2.90, p = 0.004, r = 0.75), at 3 minutes post-test (t = -5.14, p < 0.001, *dc* = 1.33), and at 1 hour post-test (Z = -3.35, p = 0.001, r = 0.87), all demonstrating large to very large effect sizes compared to pre-intervention levels. Similarly, in the PLA group, IL-1β levels were significantly elevated during the second trial at rest (Z = -2.36, p = 0.019, r = 0.61) and at 1 hour post-test (Z = -3.24, p = 0.001, r = 0.84), with large effect sizes relative to the first trial. Additionally, significant differences were noted across the three IL-1β measurements in the second trial for the PLA group (*Chi*^*2*^ = 8.22, p = 0.016, W = 0.274), though the effect size was small. No significant changes in IL-6 concentration were observed in CUR group before and after curcumin supplementation. However, the PLA group showed a significantly higher IL-6 concentration at rest during the second trial (Z = -2.58, p = 0.010, r = 0.66) compared to the first.

The PLA group exhibited significantly lower TNFα levels at rest (t = 2.74, p = 0.016, *dc* = 0.71) and at 3 minutes post-test (t = 3.12, p = 0.008, dc = 0.81) during the second trial compared to the first, with a medium and large effect sizes, respectively. Significant differences in TNFα levels were also observed across the three measurements during the second trial (*Chi*^*2*^ = 6.35, p = 0.042, W = 0.21), although the effect size was small. Between-group comparisons revealed that the CUR group had significantly higher TNFα levels at 3 minutes post-test during the second trial compared to the PLA group (t = -3.13, p = 0.004, *dc* = 0.66), with a medium effect size.

In the CUR group, significant differences in BDNF concentration were observed at rest, at 3 minutes post-test, and 1 hour post-test in both the first (*Chi*^*2*^ = 16.93, p < 0.001, W = 0.56) and second (*Chi*^*2*^ = 14.93, p < 0.001, W = 0.50) trials, showing a medium effect size. The PLA group also exhibited significant differences in BDNF concentration during the first (*Chi*^*2*^ = 13.73, p = 0.001, W = 0.46) and second (*Chi*^*2*^ = 18.34, p < 0.001, W = 0.61) trials, with medium and small effect sizes, respectively. A smaller increase in BDNF levels was observed in the CUR group at 3 minutes post-test during the second trial compared to the PLA group. Additionally, a significant difference in BDNF concentration between the CUR and PLA groups was noted at rest during the first trial (t = 2.24, p < 0.05, *dc* = 0.37).

### Blood morphology

The results of the biochemical blood morphology analysis are presented in the Table 3. In the PLA group, a statistically significant increase in lymphocyte percentages was observed at rest during the second trial compared to the first trial (Z = -2.16, p = 0.031, r = 0.56), with a large effect size. Significant differences were observed between respective values at rest, 3 min post-test, and 1 h post-test for the following variables: RGB, WBC, HGB, PLT, EOS, BASO, NEUT, and LYM in both the first and second trials for both CUR and PLA groups. Additionally, a statistically significant difference was also observed in three measurements of immature granulocytes (IGs) in the second trial (*Chi*^*2*^ = 6.41, p < 0.041, W = 0.21) in the PLA group. The blood morphology was secondary outcomes.

**Table 3:** Changes in blood morphology after supplementation of curcumin.

Variable	Group	Trial	Rest	3 min post test	1 h post test
RBC (mln/μl)	CUR	1^st^	4.94±0.29^bbb^	5.18±0.33^bbb^	4.84±0.33^bbb^
		2^nd^	4.96±0.31^bbb^	5.16±0.31^bbb^	4.78±0.32^bbb^
	PLA	1^st^	5.00±0.28^bbb^	5.17±0.33^bbb^	4.80±0.30^bbb^
		2^nd^	5.00±0.27^bbb^	5.16±0.21^bbb^	4.82±0.30^bbb^
WBC (tys/μl)	CUR	1^st^	6.11±1.85^bbb^	9.52±2.66^bbb^	6.31±2.43^bbb^
		2^nd^	5.97±1.41^bbb^	9.59±3.11^bbb^	6.12±1.86^bbb^
	PLA	1^st^	5.96±1.08^bbb^	9.24±2.11^bbb^	6.54±2.70^bbb^
		2^nd^	6.03±0.86^bbb^	8.99±2.07^bbb^	6.77±2.87^bbb^
HGB (g/dl)	CUR	1^st^	15.25±0.86^bbb^	15.97±1.03^bbb^	14.92±1.05^bbb^
		2^nd^	15.11±0.91^bbb^	15.79±0.94^bbb^	14.72±0.95^bbb^
	PLA	1^st^	15.18±0.57^bbb^	15.74±0.79^bbb^	14.68±0.64^bbb^
		2^nd^	15.16±0.53^bbb^	15.65±0.49^bbb^	14.66±0.46^bbb^
PLT (tys/μl)	CUR	1^st^	237.53±33.91^bbb^	291.60±50.81^bbb^	229.93±39.33^bbb^
		2^nd^	244.53±37.35^bbb^	298.93±37.88^bbb^	231.33±30.35^bbb^
	PLA	1^st^	258.43±32.95^bbb^	318.29±45.72^bbb^	253.50±30.00^bbb^
		2^nd^	264.86±37.63^bbb^	323.36±44.70^bbb^	253.43±38.45^bbb^
EOS (tys/μl)	CUR	1^st^	0.22±0.16^bbb^	0.25±0.20^bbb^	0.15±0.14^bbb^
		2^nd^	0.22±0.10^bbb^	0.24±0.10^bbb^	0.13±0.06^bbb^
	PLA	1^st^	0.20±0.14^bbb^	0.22±0.19^bbb^	0.12±0.09^bbb^
		2^nd^	0.20±0.11^bbb^	0.21±0.12^bbb^	0.12±0.07^bbb^
BASO (tys/μl)	CUR	1^st^	0.05±0.01^bbb^	0.08±0.02^bbb^	0.04±0.01^bbb^
		2^nd^	0.05±0.02^bbb^	0.08±0.02^bbb^	0.05±0.01^bbb^
	PLA	1^st^	0.05±0.01^bbb^	0.08±0.03^bbb^	0.05±0.02^bbb^
		2^nd^	0.05±0.02^bbb^	0.08±0.03^bbb^	0.05±0.02^bbb^
NEUT (%)	CUR	1^st^	52.65±7.79^bbb^	46.63±8.65^bbb^	66.23±7.70^bbb^
		2^nd^	53.04±7.62^bbb^	45.46±9.06^bbb^	64.97±7.73^bbb^
	PLA	1^st^	53.99±6.61^bbb^	47.72±10.23^bbb^	69.17±9.59^bbb^
		2^nd^	52.26±5.57^bbb^	46.62±6.49^bbb^	68.21±8.52^bbb^
LYM (%)	CUR	1^st^	34.11±7.74^bbb^	41.71±8.56^bbb^	22.83±6.20^bbb^
		2^nd^	33.45±7.43^bbb^	42.89±9.93^bbb^	24.57±6.65^bbb^
	PLA	1^st^	31.46±6.71^bbb^	39.99±9.26^bbb^	19.68±8.48^bbb^
		2^nd^	33.96±5.41^abbb^	41.59±6.81^bbb^	20.28±6.56^bbb^
IG (%)	CUR	1^st^	0.29±0.14	0.31±0.16	0.36±0.15
		2^nd^	0.32±0.12	0.33±0.18	0.35±0.15
	PLA	1^st^	0.24±0.10	0.25±0.08	0.31±0.14
		2^nd^	0.25±0.10^b^	0.29±0.11^b^	0.36 ±0.15^b^

Note: CUR–group supplemented with curcumin; PLA–placebo; RBC–red blood cells; WBC–white blood cells; HGB–hemoglobin; PLT–platelets; EOS—eosinophils; BASO—basophils; NEUT—neutrophils; LYM–lymphocytes; IG—immature granulocytes; significant differences: ^a^ p<0.05 vs. respective basal values before treatment; ^b^ p<0.05, ^bbb^ p<0.001 between respective values at rest, 3 min post-test and 1 h post-test

### Ratio of selected morphological indicators

The results of the ratios of selected morphological indicators are presented in Table 4. The analysis revealed statistically significant differences between the respective measurements at rest, at 3 minutes post-test, and 1 hour post-test during both first and second trials for both CUR and PLA groups. These differences were observed in neutrophil-to-lymphocyte ratio (NLR) and monocyte-to-lymphocyte ratio (MLR), both with a large effect size, as well as in platelet-to-lymphocyte ratio (PLR), which demonstrated a moderate effect size. Furthermore, in the PLA group, a significantly lower MLR (Z = 2.61, p = 0.009, r = 0.67) and a significantly lower PLR (Z = 2.44, p = 0.015, r = 0.63) were observed in the second trial at rest compared to the first trial. Between-group comparisons revealed that the CUR group exhibited a significantly lower PLR during the first trial (Z = 2.28, p = 0.023, r = 0.59) and the second trial (t = 2.17, p = 0.038, dc = 0.56) at 1 hour post-test, with large and medium effect sizes, respectively. The ratio of selected morphological indicators was secondary outcomes.

**Table 4:** Changes in ratio of selected morphological parameters after supplementation of curcumin.

Variable	Group	Trial	Rest	3 min post test	1 h post test
NLR	CUR	1^st^	1.64±0.62^bbb^	1.21±0.50 ^bbb^	3.24±1.40 ^bbb^
		2^nd^ Δ	1.72±0.66^bbb^ 0.08±0.72 (12%)	1.17±0.54 ^bbb^ -0.04±0.42 (1%)	2.90±1.26 ^bbb^ -0.28±1.14 (-2%)
	PLA	1^st^	1.83±0.61^bbb^	1.32±0.61 ^bbb^	4.22±1.83 ^bbb^
		2^nd^ Δ	1.62±0.56^bbb^ -0.21±0.45 (-8%)	1.71±0.70 ^bbb^ 0.39±1.76 (24%)	3.80±1.26 ^bbb^ -0.41±1.16 (-4%)
MLR	CUR	1^st^	0.26±0.09^bbb^	0.20±0.06 ^bbb^	0.35±0.15 ^bbb^
		2^nd^ Δ	0.27±0.14^bbb^ 0.01±0.10 (4%)^c^	0.20±0.10 ^bbb^ 0.00±0.08 (-2%)	0.31±0.11 ^bbb^ -0.05±0.10 (-9%)
	PLA	1^st^	0.33±0.13^bbb^	0.23±0.09 ^bbb^	0.42±0.20 ^bbb^
		2^nd^ Δ	0.29±0.13^aabbb^ -0.04±0.06 (-10%)^c^	0.31±0.07 ^bbb^ 0.08±0.40 (39%)	0.40±0.13 ^bbb^ -0.02±0.16 (4%)
PLR	CUR	1^st^	123.79±41.64^bbb^	80.47±27.24 ^bbb^	186.7±86.88 ^bbbc^
		2^nd^ Δ	133.83±46.92^bbb^ 10.04±25.12 (10%)^c^	84.89±39.61 ^bbb^ 4.42±20.29 (4%)	172.51±44.63 ^bbbc^ -16.17±89.61 (-16%)
	PLA	1^st^	146.25±41.13^bbb^	96.39±42.78 ^bbb^	234.87±70.32 ^bbbc^
		2^nd^ Δ	134.29±32.17^abbb^ -11.96±17.77 (-7%)^c^	139.62±178.7 ^bbb^ 43.23±181.44 (56%)	209.37±48.15 ^bbbc^ -25.50±51.80 (-8%)

Note: CUR–group supplemented with curcumin; PLA–placebo; NLR–neutrophil-to-lymphocyte ratio; MLR–monocyte-to-lymphocyte ratio, PLR–platelet-to-lymphocyte ratio; Δ–difference between 2^nd^ and 1^st^ measurements; significant differences: ^a^ p<0.05, ^aa^ p<0.01, ^aaa^ p<0.001 vs. respective basal values before treatment; ^b^ p<0.05, ^bb^ p<0.01, ^bbb^ p<0.001 between respective values at rest, 3 min post-test and 1 h post-test; ^c^ p<0.05 between groups (CUR vs. PLA)

## Discussion

The aim of this study was to determine the effect of a 6-week supplementation with curcumin at a dose of 2g per day on reducing inflammation, BDNF levels, and blood morphology in amateur middle-aged long-distance runners during the preparatory training period.

High-intensity interval training, resistance training, and mountain running are known to induce skeletal muscle damage due to physical exertion, leading to muscle structural disruptions and elevated levels of inflammatory cytokines [37, 38]. Endurance training, in particular, is associated with a gradual increase in inflammation and muscle soreness, which can ultimately result in overuse injuries [39]. In response to inflammation, leukocytes release cytokines such as IL-1β, IL-6, and TNF-α, with significant changes in these cytokine levels being observed after endurance exercises [40]. IL-6, a key mediator, promotes the expression of the acute phase protein gene (CRP), while IL-1 enhances this effect [41]. Plasma CRP levels can rise from approximately 1 μg/ml to over 500 μg/ml within 24–72 hours following severe tissue damage [42].

In the present study, curcumin supplementation was found to have a nonsignificant effect on reducing IL-6 levels, with only a 4% decrease observed, while the PLA group showed a significant 18% increase. Considering that the promoter region of the IL-6 coding gene contains binding sites for *NF-κ* B, C/ *EBP β*, and c-Jun [43], curcumin’s therapeutic potential may be associated with its inhibitory effect on *NF-κ* B, thereby modulating cytokine expression [44]. However, the optimal dosage of curcumin required to effectively reduce pro-inflammatory IL-6 levels remains uncertain [38].

Another indicator analyzed in this study was the cytokine TNFα. Although scientific evidence supports the potential of curcumin in reducing post-exercise inflammation [45], our findings did not reveal such an effect. Similarly, results obtained by Basham et al. [15], who assessed the effect of curcumin supplementation at a dose of 1.5 g/day for 28 days on athletes undergoing stress tests leading to skeletal muscle damage, did not confirm any significant effect of this supplement on the natural inflammatory response following exercise.

Pro-inflammatory cytokines, such as TNF-α and IL-1β, which increase following prolonged and/or intensive exercise, are known to play roles in the acute phase response and cell proliferation [46]. After supplementation, significantly higher resting IL-1β concentration were observed in the CUR group, although the relative increase in interleukin levels was lower in the supplemented group (8%) compared to the PLA group (15%). Despite this, no beneficial effects on post-exercise IL-1β concentrations were detected following curcumin supplementation.

CRP levels demonstrated considerable inter-individual variability, with both groups showing increased concentrations during the second trial. However, the increase in resting CRP was 4.4 times lower in the CUR group, and immediately post-exercise and 1 hour into recovery, the increases were 1.8 and 1.9 times lower, respectively. Previous study indicated that curcumin supplementation for 4 days at a dose of 2 g/day had a non-significant effect on post-exercise changes in CRP concentration [27].

BDNF is a neurotrophin that is widely distributed throughout the nervous system [47], and its release can be triggered by endurance exercises [48]. BDNF levels typically rise significantly following intense physical activity, and improved cardiorespiratory fitness may further enhance this increase [49]. However, the effects of curcumin supplementation on BDNF levels in endurance athletes remain largely unexplored.

In assessing BDNF levels in long-distance runners, significantly different resting BDNF levels were observed between the CUR and PLA groups during the first trial. Although no statistically significant changes in BDNF levels were found in either group, the CUR group exhibited a 21% increase in BDNF after 6 weeks of supplementation at rest, while a 5% decrease was observed in the PLA group. Exercise did not result in a significant elevation in serum BDNF levels.

There is limited information on the effects of curcumin on blood cell parameters in humans, especially among long-distance runners. In the present study, no significant changes in selected hematological parameters (RBC, WBC, HGB, PLT, EOS, BASO, % NEUT, % IG) were found between the CUR and PLA groups. The observed post-exercise variations in hematological parameters in both groups may be attributable to the modulation of these levels by physical activity. The results obtained do not provide conclusive evidence regarding the effect of curcumin on hematological parameters, consistent with findings from other studies [50, 51]. Nevertheless future research should consider conducting more extensive studies on long-distance runners to better understand the influence of curcumin on blood rheological parameters.

Hematological ratios such as NLR, MLR, and PLR can be used to assess systemic inflammation and are often employed in the diagnosis of various conditions [52]. Elevated NLR levels have been observed in thyroid diseases, gastrointestinal diseases, diabetes, and SARS-CoV-2 infections, while increased PLR levels have been associated with cancer, diabetes, and irritable bowel syndrome. MLR is also suggested as a marker in cancer and gastrointestinal disorders [53, 54]. These markers could potentially be beneficial for monitoring the health status of athletes [55].

A study by Schlagheck et al. [56] found that endurance exercise resulted in a greater increase in NLR compared to strength training, likely due to heightened mobilization of immune cells in response to strenuous exercise. NLR reflects changes in the two primary immune cell populations affected by exercise (neutrophils and lymphocytes), whereas PLR may not be sensitive enough to detect exercise-induced alterations in cellular immune homeostasis, despite previous studies reporting changes in this ratio following intense exercise [57]. However, the results of this study do not clearly support the use of NLR, MLR, or PLR as reliable markers for diagnosing exercise-induced inflammation.

### Study limitation

This study has several limitations. It is noteworthy that the participants did not undergo analysis for curcumin metabolites in their blood serum and urine. Furthermore, the dietary intake of the participants, especially concerning curcumin content, was not monitored or controlled. Dietary intake can significantly influence the levels of curcumin and its metabolites in the body, and thus, the potential effects of curcumin supplementation. Due to the failure to achieve the expected effect size in five tests, these results should be interpreted with caution. In future studies, increasing the sample size should be considered to improve the reliability of the findings.

## Conclusion

Despite the observed trends toward lower increases in acute phase proteins and pro-inflammatory cytokines (IL-6 and IL-1β), our research results did not validate the hypothesis that 6-week curcumin supplementation significantly mitigates exercise-induced inflammation in the studied cohort of male long-distance runners. Regarding BDNF concentration, a noticeable trend towards an elevation in the level of this neurotrophin was observed following 6 weeks of supplementation. However, no statistically significant differences in BDNF levels were identified. Additionally, no significant changes in blood count were detected before and after supplementation.

Our findings suggest a potential trend toward reduced inflammation and elevated BDNF levels with curcumin supplementation, but these changes were not statistically significant in the studied population. Further research is needed to validate the potential benefits of curcumin supplementation during the preparatory period of training cycles for long-distance runners. This will help to elucidate the role of curcumin supplementation in exercise-induced inflammation and neurotrophin regulation. Future studies should also account for dietary intake to more comprehensively evaluate the effects of curcumin supplementation in this population.

## Supporting information

S1 Checklist CONSORT 2010 checklist of information to include when reporting a randomised trial*. (DOC)

S1 Data (XLSX)

S1 File (DOCX)

S2 File (DOCX)
