# Supplementary material for: Does curcumin supplementation affect inflammation, blood count and serum brain-derived neurotropic factor concentration in amateur long-distance runners?
Source: PLoS One. 2025 Jan 14;20(1):e0317446. doi: 10.1371/journal.pone.0317446 (PMC11731706; doi:10.1371/journal.pone.0317446)
Supplement: S1 File — (DOCX) [file pone.0317446.s003.docx]

**Protokół badania -** **Wpływ suplementacji kurkuminą na stres oksydacyjny, stan zapalny i uszkodzenie mięśni wywołane wysiłkiem fizycznym**

**Badanie zostaną przeprowadzone w dwóch etapach:**

- w pierwszym etapie wszyscy badani i zostaną poddani ocenie podstawowych parametrów antropometrycznych z wykorzystaniem InBody Data Management System. Następnie wszyscy badani wykonają test biegowy o stopniowo narastającym obciążeniu na bieżni ruchomej (Cosmed, Niemcy) w celu oceny wydolności tlenowej. W czasie testu szybkość biegu będzie wzrastała, co 3 min o 2km/h, aż do osiągnięcia prędkości biegu 14 km/h, a następnie zwiększany będzie kąt nachylenie bieżni do podłoża, aż do osiągnięcia indywidualnie maksymalnego obciążenia organizmu pracą mięśniową. Podczas biegu będzie dokonywany stały pomiar poboru tlenu przy użyciu analizatora gazowego (Oxycon, Jaeger, Niemcy) oraz częstość skurczów serca przy użyciu sport-testera Polar-3500PE (Finlandia). Krew do badań biochemicznych będzie pobierana 3-krotnie tj. przed rozpoczęciem testu wysiłkowego, 3-5 min. po jego zakończeniu i w 1 godz. restytucji powysiłkowej. Następnie badani zostaną losowo podzieleni na dwie grupy: suplementowaną kurkuminą i placebo.

-w drugim etapie badań (po upływie 6 tygodni od rozpoczęcia przyjmowania suplementu lub placebo) badani ponownie zostaną poddani ocenie podstawowych parametrów antropometrycznych oraz wykonają test biegowy jw. Krew do badań biochemicznych będzie pobierana 3-krotnie tj. przed rozpoczęciem testu wysiłkowego, 3-5 min. po jego zakończeniu i w 1 godz. restytucji powysiłkowej.

**Suplmentacja**

Badani przez okres 6 tygodni będą przyjmować suplement diety lub placebo. Badani będą zażywać 2 razy dziennie po 2 kapsułki ekstraktu z kurkuminy z dodatkiem piperyny (*Kurkumina 500 max,* *Magiczny Ogród*) przed posiłkiem tj. przed śniadaniem i przed kolacją, popijając wodą, przez okres 6 tygodni. Zgodnie z informacją producenta zawartość kapsułki suplementu stanowią: ekstrakt z ostryżu długiego standaryzowanego na zawartość 95% kurkuminy (*500 mg*) oraz ekstrakt z pieprzu czarnego 95% (*10 mg*), składnik otoczki (żelatyna, woda oczyszczona 13-17%).

**Placebo**

Zgodnie z informacją producenta zawartość kapsułki suplementu stanowią: skrobia kukurydziana (*400 mg*), barwnik: ryboflawina (*10 mg*), składnik otoczki (żelatyna, woda oczyszczona 13-17%).

**Kryteria włączenia i wyłączenia z badań**

Warunkiem doboru zawodników do badań będzie spełnienie następujących kryteriów:

- dobrowolna zgoda na badanie,

- staż treningu sportowego ukierunkowanego na długie dystanse minimum 3 lata,

- osoby pełnoletnie,

- dobry stan zdrowia i brak przeciwwskazań do wykonania próby wysiłkowej,

- nieprzyjmowanie przez badanych suplementów o właściwościach antyoksydacyjnych i/lub leków przeciwzapalnych w okresie poprzedzającym (co najmniej 4 tygodnie) badania oraz w czasie ich realizacji.

Podstawowym kryterium wyłączenia z badań będzie:

-brak zgody na udział w badaniach,

-przeciwwskazania zdrowotne potwierdzone zaświadczeniem lekarskim,

- niedyspozycyjność w dniu badania (np. kontuzja, zdiagnozowany stan zapalny),

- przyjmowanie przez badanego suplementów o właściwościach antyoksydacyjnych i/lub leków przeciwzapalnych w okresie poprzedzającym (co najmniej 4 tygodnie) badania oraz
w czasie ich realizacji,

- staż treningowy mniejszy niż 3 lata.

**Study protocol - Effect of curcumin supplementation on oxidative stress, inflammation and exercise-induced muscle damage**

**The study will be conducted in two phases:**

- in the first phase all subjects and will undergo assessment of body composition (InBody Data Management System). Then all participants underwent an incremental treadmill running test (a Cosmed treadmill, Germany) while connected to a breath-by-breath gas analyzer (MetaLyzer 3B-R2, Leipzig, Germany) to determine maximal oxygen uptake (VO_2_max) twice, i.e., before the start (1st trial) and after 6 weeks of supplementation with either placebo or curcumin (2nd trial). Heart rate was monitored using a Polar-3500PE sports-tester (Finland). The treadmill speed was increased every 3 minutes by 2 km/h until a running speed of 14 km/h was reached; thereafter, the tilt angle was progressively increased by 2.5º every 3 minutes until exhaustion.

Blood for biochemical tests will be collected 3 times, i.e., before the start of the exercise test, 3-5 minutes after its completion and at 1-hour post-exercise restitution. The subjects will then be randomly divided into two groups: curcumin supplemented and placebo.
-in the second phase of the study (6 weeks after starting the supplement or placebo), the subjects will again undergo assessment of baseline body composition and perform a running test as above. Blood for biochemical tests will be collected 3 times, i.e., before the start of the exercise test, 3-5 minutes after its completion and at 1-hour post-exercise restitution.

**Exclusion and inclusion criteria**

Exclusion criteria included tobacco use, alcohol consumption, intake of any medications, non-steroidal anti-inflammatory drugs (NSAID’s), or dietary supplements in the four weeks prior to the study. Inclusion criteria were being an adult male with a minimum of three years of training experience.

**Supplement**

Supplements were administered in the form of soft gelatinous capsules (Nanga, Złotów, Poland) at a dose of 2 g curcumin extract daily for six weeks. This involved taking 2 capsules after breakfast and dinner with a glass of water. Each supplement capsule consisted of a common turmeric extract standardized to contain 95% of curcumin (500 mg), black pepper extract standardized to contain 95% piperine (10 mg), and a shell composed of gelatine and purified water (13-17%).

**Placebo**

The placebo contained corn-starch (400 mg), riboflavin pigment (10 mg), and a shell composed of gelatine and purified water (13-17%).
