# Supplementary material for: Does curcumin supplementation affect inflammation, blood count and serum brain-derived neurotropic factor concentration in amateur long-distance runners?
Source: PLoS One. 2025 Jan 14;20(1):e0317446. doi: 10.1371/journal.pone.0317446 (PMC11731706; doi:10.1371/journal.pone.0317446)
Supplement: S2 File — (DOCX) [file pone.0317446.s004.docx]

**To the Bioethics Committee of the Jerzy Kukuczka Academy of Physical Education in Katowice, Poland**

**RESEARCH APPLICATION**

Research topic:

**Effect of curcumin supplementation on oxidative stress, inflammation and exercise-induced muscle damage**

This is the original submission to the bioethics committee with sensitive data removed and the part not related to the study protocol.

**b. methodology**

**The study will be conducted in two phases:**

- in the first phase all subjects and will undergo assessment of body composition (InBody Data Management System). Then all participants underwent an incremental treadmill running test (a Cosmed treadmill, Germany) while connected to a breath-by-breath gas analyzer (MetaLyzer 3B-R2, Leipzig, Germany) to determine maximal oxygen uptake (VO_2_max) twice, i.e., before the start (1st trial) and after 6 weeks of supplementation with either placebo or curcumin (2nd trial). Heart rate was monitored using a Polar-3500PE sports-tester (Finland). The treadmill speed was increased every 3 minutes by 2 km/h until a running speed of 14 km/h was reached; thereafter, the tilt angle was progressively increased by 2.5º every 3 minutes until exhaustion.

Blood for biochemical tests will be collected 3 times, i.e., before the start of the exercise test, 3-5 minutes after its completion and at 1-hour post-exercise restitution. The subjects will then be randomly divided into two groups: curcumin supplemented and placebo.

-in the second phase of the study (6 weeks after starting the supplement or placebo), the subjects will again undergo assessment of baseline body composition and perform a running test as above. Blood for biochemical tests will be collected 3 times, i.e., before the start of the exercise test, 3-5 minutes after its completion and at 1-hour post-exercise restitution.

**Exclusion and inclusion criteria**

Exclusion criteria included tobacco use, alcohol consumption, intake of any medications, non-steroidal anti-inflammatory drugs (NSAID’s), or dietary supplements in the four weeks prior to the study. Inclusion criteria were being an adult male with a minimum of three years of training experience.

**Supplement**

Supplements were administered in the form of soft gelatinous capsules (Nanga, Złotów, Poland) at a dose of 2 g curcumin extract daily for six weeks. This involved taking 2 capsules after breakfast and dinner with a glass of water. Each supplement capsule consisted of a common turmeric extract standardized to contain 95% of curcumin (500 mg), black pepper extract standardized to contain 95% piperine (10 mg), and a shell composed of gelatine and purified water (13-17%).

**Placebo**

The placebo contained corn-starch (400 mg), riboflavin pigment (10 mg), and a shell composed of gelatine and purified water (13-17%).
